# Sensor-Based Indoor Fire Forecasting Using Transformer Encoder

**DOI:** 10.3390/s24072379

**Published:** 2024-04-08

**Authors:** Young-Seob Jeong, JunHa Hwang, SeungDong Lee, Goodwill Erasmo Ndomba, Youngjin Kim, Jeung-Im Kim

**Affiliations:** 1 Department of Computer Engineering, Chungbuk National University, Cheongju 28644, Republic of Korea; ysjay@chungbuk.ac.kr (Y.-S.J.); jhhwghg9911@chungbuk.ac.kr (J.H.); sdlee130@chungbuk.ac.kr (S.L.); goodwillndomba22@chungbuk.ac.kr (G.E.N.); 2 Frugal Solution, Daejeon 34126, Republic of Korea; bada0179@fscom.kr; 3 School of Nursing, College of Medicine, Soonchunhyang University, Cheonan 31151, Republic of Korea

**Keywords:** fire detection, deep learning, transformer, multiple sensors, time-series data

## Abstract

Indoor fires may cause casualties and property damage, so it is important to develop a system that predicts fires in advance. There have been studies to predict potential fires using sensor values, and they mostly exploited machine learning models or recurrent neural networks. In this paper, we propose a stack of Transformer encoders for fire prediction using multiple sensors. Our model takes the time-series values collected from the sensors as input, and predicts the potential fire based on the sequential patterns underlying the time-series data. We compared our model with traditional machine learning models and recurrent neural networks on two datasets. For a simple dataset, we found that the machine learning models are better than ours, whereas our model gave better performance for a complex dataset. This implies that our model has a greater potential for real-world applications that probably have complex patterns and scenarios.

## 1. Introduction

There is an average of 358,300 home-based fires every year in the U.S. according to the Center for Disease Control, the U.S. Fire Administration, and the National Fire Protection Association (NFPA). Indoor fires cause many casualties and property damage, so it is important to prevent fire accidents in advance. Previous studies of the indoor fire prediction task can be divided into two groups: sensor-based and vision-based. The vision-based studies exploit video clips or image samples to detect fires or any clues of fire, whereas the sensor-based studies utilize time-series values collected from multiple sensors (e.g., CO_2_, humidity, and temperature). The images and video clips are usually much larger than the sensor values, so the sensor-based approach is preferable if it is given a limited computational resource or Internet bandwidth.

There have been sensor-based studies for indoor fire forecasting using machine learning (ML) models. The ML models require intensive feature engineering, so deep learning (DL) models have become more preferable. In particular, recurrent neural networks (RNNs) using long short-term memory (LSTM) [1] or Gated Recurrent Unit (GRU) [2] cells allow to effectively comprehend long-term dependencies within time-series values of multiple sensors.

Since Transformer [3] has appeared, many Transformer-based language models have shown impressive power in learning linguistic patterns within documents. In particular, the Transformer encoder-based models (e.g., Bidirectional Encoder Representations from Transformer (BERT) [4]) are used not only for language comprehension tasks but also for other domains such as object detection in images [5] and classification using time-series sensor data [6]. The multi-head self-attention mechanism of the Transformer allows it to better grasp the sequential patterns within the time-series data and contributes to performance improvement in down-stream tasks.

In this paper, we propose a new way of fire prediction using multiple sensors. The task is defined as the prediction of potential fires in advance given a certain amount of collected sensor data. To tackle the task, we design a stack of Transformer encoders where the Transformer architecture is known to be effective in analyzing sequence data. Our first contribution is that, as far as we know, this is the first study that employs the Transformer encoders to the fire prediction task. Our model takes the time-series values obtained from the sensors as input, and predicts fire accidents in advance. Our second contribution is that, by empirical results with two publicly available datasets, we demonstrate that our model has a strong potential in a complex fire prediction task.

## 2. Related Work

There are lots of studies that have utilized images or video clips for fire prediction. Such a vision-based approach takes the benefit from sufficient information beneath the images, but these studies have a common practical issue that they require a high-resolution camera and a sufficient amount of computational power. That is, big images are required to obtain better detection performance, and it takes a long time or requires expensive machines to train or run machine learning (ML) models with the big images. Therefore, using many cheaper sensors is a reasonable alternative way.

There are studies that have exploited multiple sensors for fire prediction, and they mostly employed the machine learning (ML) or data-driven models. In [7], a data-fusion based on Dempster–Shafer theory was used to aggregate smoke, temperature, and light sensor values, and achieved 98% of accuracy. Chen et al. [8] proposed a fast and cost-effective indoor fire alarm system using support vector machines (SVMs) [9]. They employed carbon monoxide, smoke, temperature and humidity sensor values, and accomplished 99.8% of F1 scores. Jana and Shome [10] proposed an ensemble of ML models such as logistic regression, SVM, decision tree, and Naive Bayes, and obtained 97.52% precision. Dampage et al. [11] utilized ML models to detect forest fires at initial stage using a wireless sensor network.

Even though the ML models exhibited quite successful performance on the fire prediction task, deep learning (DL) models have become more preferable for their superior robustness and performance without a heavy manual feature engineering. In [12], given CO_2_, smoke, and temperature sensor values, they obtained 99.4% accuracy using a back-propagation neural network (BPNN). Nakip et al. [13] proposed a recurrent trend predictive neural network (rTPNN) for multi-sensor fire detection, where the sensors include temperature, smoke, carbon monoxide, carbon dioxide, and oxygen sensors. Li et al. [14] utilized a temporal convolutional network (TCN) to extract features, and generated prediction results using the SVM classifier. Jesubalan et al. [15] designed a learning-based mechanism for forest fire prediction using deep learning models. Liu et al. [16] adopted long short-term memory (LSTM) [1] to analyze sequences of temperature, smoke, carbon dioxide, and carbon monoxide, and achieved 97% F1 score.

Since Transformer [3] of the natural language processing (NLP) field has appeared, many of its variants including ChatGPT (https://chat.openai.com) have achieved state-of-the-art (SOTA) performance in various fields with different data types such as images, speeches, texts, and sensors. There are a few studies that have applied the self-attention mechanism of Transformer to fire prediction, but they have focused only on the image or video data. For example, FireFormer is a Transformer-based architecture dedicated to forest fire detection using surveillance cameras [17]. In Ref. [18], a Transformer-based model for the detection of fire in videos was proposed, and showed that their model outperformed previous convolutional neural network (CNN) models.

In this paper, we propose a new model that is essentially a stack of Transformer encoders for fire prediction. It takes sequences of multiple sensor values, and predicts potential fire accidents. The ‘multi-head self-attention’ mechanism of Transformer captures various relations of all possible pairs in the sequence of sensor values, and the ‘multi-layer’ architecture allows it to comprehend high-level semantics and syntactic patterns. This enables it to better learn complex sequential patterns of the given sequences, contributing to performance improvements. As far as we know, this is the first study that proposes a Transformer-based architecture for fire prediction using multiple sensor values.

## 3. Method

### 3.1. Problem

The task of this paper is to predict fires in advance. Given a certain amount of collected data of multiple sensors, we predict whether the fire will break out in several minutes. Suppose we have a database (DB) system that collects time-series values from *S* sensors, where the sensor values are sampled using a particular sampling rate *R* (e.g., every 2 s). The collected dataset $D\in R^{|D|\times S}$, where |D| is the total number of instances (i.e., the total number of steps) within the dataset.

**Figure 1:**
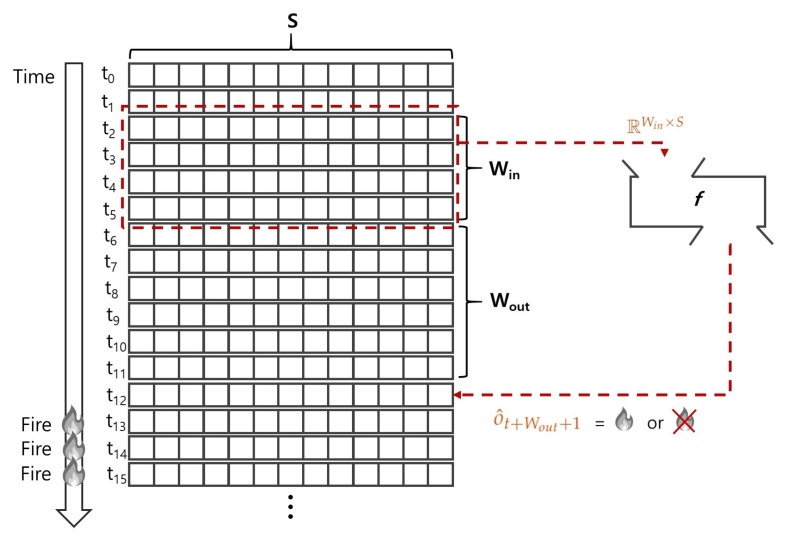
Overview of the method, where the current step is $t_{5}$, the input window size $W_{in}=4$, and the output window size $W_{out}=6$.

Figure 1 describes the input and output of the model *M*. Given the current step $t_{5}$, the model takes the instances of window size $W_{in}$ as input (i.e., $R^{W_{in}\times S}$). That is, the input matrix consists of [$f_{t_{2}}$;$f_{t_{3}}$;$f_{t_{4}}$;$f_{t_{5}}$] where $f_{t\cdot }$ indicates the *S*-dimensional feature vector at the step t·. The feature vector does not contain temporal information or any time-related feature values (e.g., timestamps). The model generates a prediction label ${\hat{o}}_{12}$ (e.g., ‘fire’, ‘normal’) of the step $t_{12}$ which is $W_{out}$ steps ahead of the current step $t_{5}$, where the $W_{out}$ is the output window size. The prediction label ${\hat{o}}_{12}$ indicates whether the fire breaks out after $W_{out}$ steps from the current step $t_{5}$. The model fails when its prediction ${\hat{o}}_{12}$ is different from the actual label $o_{12}$. More formally, the output (i.e., prediction label) generated from classifier *f* can be represented as Equation (1), where the input matrix $M_{t-W_{in}+1:t}\in R^{W_{in}\times S}$:

(1) $${\hat{o}}_{t+W_{out}+1}=f\left(M_{t-W_{in}+1:t};W_{out}\right)$$

### 3.2. Solution

Transformer [3] was originally designed for machine translation between languages. It has an encoder–decoder architecture, where the encoder converts a given input sequence into real-number vectors of distributed representation, and the decoder generates an output sequence based on the results of the encoder. The Transformer decoder was employed in the Generative Pre-trained Transformer (GPT) series [19,20,21], where their most well-known product is ChatGPT. On the other hand, the Transformer encoder was adopted in Bidirectional Encoder Representations from Transformer (BERT) [4] and its many variants. The encoder is designed to learn embeddings for various predictive tasks, so we take the Transformer encoder to address the task of fire prediction.

**Figure 2:**
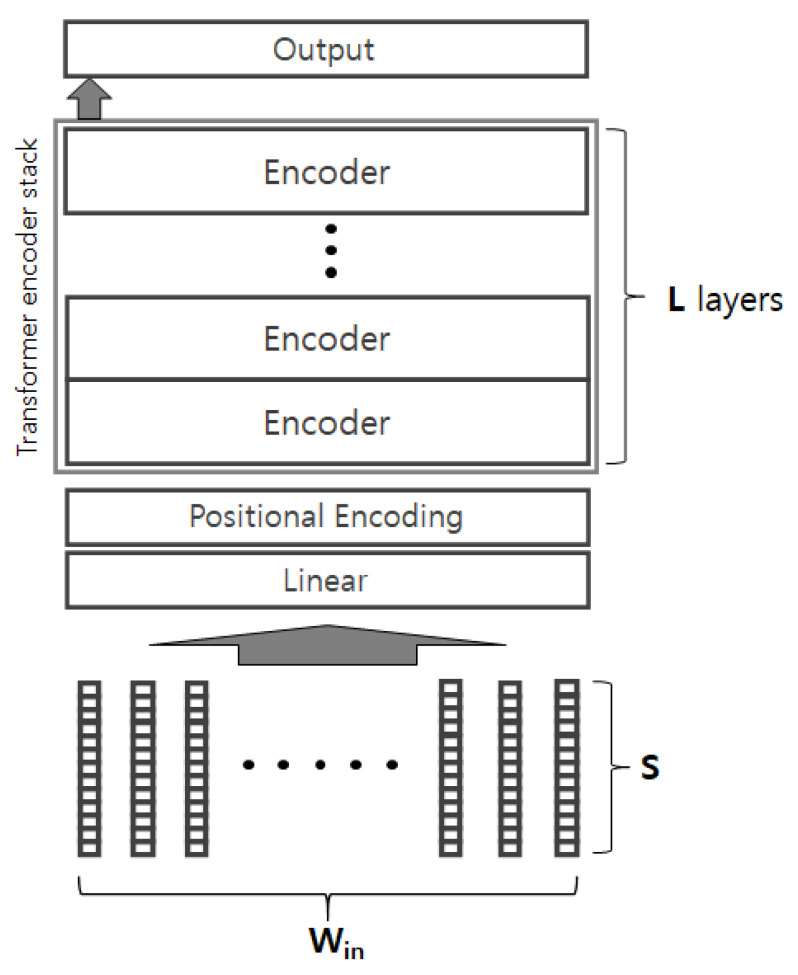
Model structure.

Figure 2 depicts the structure of our encoder-based model. The model takes a sequence of *S*-dimensional vectors $R^{W_{in}\times S}$ as an input, and the *S*-dimensional vectors are firstly mapped to another *h*-dimensional representation space through the linear layer. Note that $R^{W_{in}\times S}$ is the input matrix in the Equation (1), where the $W_{in}$ is the input window size; please refer to Figure 1 for an example of window size. The positional encoding (PE) of the Transformer is applied to the vectors, and they are passed to the stack of encoders. If we regard the PE as a function, then it takes a position index as an input and generates a real-number vector that represents the positional information. The generated real-number vector (i.e., position vector) usually takes the same shape of the input embedding vectors; in this paper, the position vector will be the *h*-dimensional vector so that it can be combined with the *h*-dimensional embedding vector generated by the linear layer through arbitrary function (e.g., element-wise addition). The PE allows it to consider the positional information of the sensor values so that it grasps the sequential patterns of the sequence. The Transformer encoder has a ‘multi-head self-attention’ mechanism, where the self-attention analyzes pair-wise relations between any pairs of a given sequence, and the multi-head enables it to analyze various types of relations. Suppose the encoder takes *h*-dimensional vector as an input, then the encoder generates the *h*-dimensional vector as an output. This allows a stacked encoder architecture. The number of encoder layers *L* depends on the task complexity. The left-most representation generated from the last encoder layer is finally delivered to the output layer for the prediction. The output layer dimension depends on the number of classes. The output layer can be followed by a Softmax function for multi-class classification. The model structure is basically similar to the original Transformer encoder. The major difference is that our model takes the real-number feature vectors, so it does not have any embedding layer (i.e., look-up table) and just linearly converts the vectors into *h*-dimensional vectors. That is, the embedding layer of original Transformer takes tokens (i.e., categorical values) as input, whereas our model takes sensor values (i.e., numerical values) as input, and the linear layer projects the sensor values into the *h*-dimensional space. Another difference is that our model is designed for the classification task, so it has the output layer on top of the encoder stack.

One may ask why do we need to choose the Transformer encoder for the fire prediction using the time-series data (i.e., sequences of multiple sensor values). Instead of the Transformer, there are some alternatives for the sequence analysis; for example, long short-term memory (LSTM) [1] and Gated Recurrent Unit (GRU) [2] are known to be effective in analyzing sequential patterns, so they probably have a strong potential in the fire prediction task. Although these alternatives have shown quite successful performance on tasks of sequential data, the Transformer exhibited better performance in recent studies [22,23,24,25]. The reason is the multi-head self-attention mechanism of the Transformer, which enables to better comprehend contextual information in the input sequence and to model long-term dependencies. Some studies report that the RNN models (e.g., LSTM and GRU) outperform the Transformer [26]. However, we found that there are no studies that have exploited Transformer for the fire forecasting task, so we conduct experiments to compare the Transformer with other models, including RNNs.

## 4. Experiment

### 4.1. Dataset

Two datasets are used for experiments in this paper. The first dataset is obtained from NIST report of test FR 4016 (https://www.nist.gov/el/nist-report-test-fr-4016, accessed on 4 April 2024), where it provides 96∼130 feature values (i.e., 96≤S≤130) collected in a manufactured house and a two-story house. The features correspond to various sensors of temperature, CO, CO_2_, O_2_, smoke, etc. The ‘NIST’ dataset contains several files, each of which has feature values collected from a distinct room. We utilized only 96 features that commonly appeared in all files. We observed that five files (e.g., sdc01, sdc03, sdc04, sdc05, and sdc06) have different sampling rates (e.g., 5 s), so we discarded them. The number of remaining files is 22, and their sampling rate $R_{NIST}$ is 2 s. The ‘TIME’ column of the dataset indicates the relative time based on the time of fire; for example, ‘TIME = −3’ means the corresponding instance is obtained 3 s before the fire, whereas ‘TIME = 5’ represents that the instance is obtained 5 s after the fire. We generated the ‘label’ column, and set o= to ‘normal’ for instances if TIME <0, whereas o= ‘fire’ when TIME ≥0; the task of the NIST dataset is binary classification. Please refer to the official web page for more details.

The second dataset, denoted as the ‘Pascal’ dataset in this paper, is collected in a standard EN 54 test room [27]. It contains a single file that has 16 columns, where the ‘ternary label’ ∈ {background(normal), nuisance, fire} is used as a label; the task of the Pascal dataset is ternary classification. The ‘nuisance’ indicates the activation of the fire alarm, caused by mechanical failure, malfunction, improper installation, lack of proper maintenance, or any other reason. It has 11 features (i.e., S=11) of several different sensors, including CO, CO_2_, H_2_, humidity, temperature, etc. We found that the sampling rate $R_{Pascal}$ is 1.11200 s in average. Please refer to the official web page (https://data.mendeley.com/datasets/npk2zcm85h/1, accessed on 4 April 2024) of the dataset for more details.

For experiments, we defined the input as *S*-dimensional feature vectors for 60 s. The sampling rates of NIST and Pascal datasets are 2 s and nearly 1 s, respectively, so the $W_{in}$ is 30 and 60 for the NIST and Pascal datasets, respectively. The output is defined as a label after 300 s, so $W_{out}$ is 150 and 300 for the NIST and Pascal datasets, respectively. This setting is based on the report that the first 5 min is important to prevent severe fire damage [28]; it will be greatly helpful if we can secure another 5 min. From the two original datasets, we randomly extracted samples, and those are denoted as $D_{NIST}$ and $D_{Pascal}$, where $D_{\cdot }=\{\left(F_{i},o_{i}\right):1\leq i\leq |D_{\cdot }\left|\right\}$. The input matrix of *i*-th sample $F_{i}=R^{W_{in}\times S}$. The *i*-th output $o_{i}\in${fire, normal} for the $D_{NIST}$, whereas $o_{i}\in$ {fire, nuisance, normal} for the $D_{Pascal}$. Table 1 summarizes the statistics of the sampled datasets. The models learn from the training samples, and make a prediction for each test sample. For all experiments, we took 10% of the training set as a validation set for a grid searching.

### 4.2. Result

We compared our model with other machine learning (ML) models such as support vector machine (SVM) [9] and random forest (RF) [29]. For these models, we flattened the matrix $R^{W_{in}\times S}$ into *K*-dimensional vector where $K=W_{in}\times S$. By the grid searching, we found no performance variation with different hyper-parameter settings of the SVM, so we followed the default setting (e.g., 1.0 for the regularization parameter C, RBF kernel with a scaled coefficient, and 1 × 10^−3^ tolerance for the stopping criterion). On the other hand, the RM gave the best performance when the number of estimators was 200, and the other remaining hyper-parameters followed the default setting (e.g., gini criterion, unlimited depth, and minimum samples of a leaf is 1). We also conducted experiments with multi-layered GRU, where the dimension of the hidden layer was 128. We used ‘Scikit-learn’ and ‘PyTorch’ packages to implement machine learning models and deep learning models, respectively.

Table 2 describes the performance of the models on $D_{NIST}$, where the metric is the overall accuracy, false positive rate (FPR), true positive rate (TPR), and TPR-FPR. All results are averages of three independent runs. For our model, the Transformer encoder stack, we varied the number of encoder layers from 1 to 8. The dimension of hidden representation is 32, the embedding dimension is 128, and the number of heads is 4. The results in Table 2 demonstrate that our models generally outperform the ML models and the GRU. This is consistent with previous studies that have shown performance improvement by Transformers; the multi-head self-attention mechanism of Transformer allowed to better comprehend the sequential patterns underlying the time-series sensor values. In other words, the multi-head self-attention mechanism extracts pair-wise relations from the sequences, and the multi-layered encoder grasps high-level relations or semantics, contributing to the performance improvement. Our model gave the best results when the number of layers was four. For fair comparison, we made the GRU have the same number of layers. We employed the cross-entropy loss for training GRU and Transformer encoder stacks. Equation (2) represents the cross-entropy loss, where $p_{\cdot }$ and $q_{\cdot }$ indicate a predicted distribution and one-hot correct vector (i.e., gold vector), respectively. The number of epochs is 15 for 4-layered GRU, and 40∼55 for Transformer encoder stacks. We employed the Adam optimizer [30] with an initial learning rate 0.0001, and early-stop strategy with five patience steps. The size of mini batches was 32. The four-layered GRU has 384 K trainable parameters, while the Transformer encoder stack of four layers has 312 K trainable parameters:

(2) $$L=-\sum_{k=1}^{h}q_{k}\log\left(p_{k}\right)$$

Table 3 summarizes the experimental results on $D_{Pascal}$. In this table, the RF gives the best performance, and the two-layered GRU shows a comparable performance to the Transformer encoder stacks. The reason for this result is the complexity of the dataset. That is, the feature dimensions of $D_{NIST}$ and $D_{Pascal}$ are 96 and 11, respectively, meaning that the $D_{NIST}$ has more complex sequential patterns. Such a complexity gap between the datasets caused the performance gap; the overall performance on $D_{NIST}$ is much lower than that of $D_{Pascal}$. The Transformer encoder stack gave its best performance when the number of layers was two, which implies that the two-layered structure was enough to analyze the sequential patterns of $D_{Pascal}$. In other words, the complexity of $D_{Pascal}$ is relatively low, so the shallow structure (i.e., two-layered) was enough to analyze such simple sequential patterns of the dataset. To summarize, for a simple and easy task, the ML models may outperform the deep learning (DL) models, including Transformers. On the other hand, the Transformer-based model has a strong potential for the dataset of high complexity.

We also examined the inference time of the proposed model because it would not be applicable to real-world applications if it takes too long to predict potential fires. We found that the eight-layered Transformer encoder took 0.54 s for 503 data instances, meaning that it will work for predicting fires in five minutes.

## 5. Conclusions

We introduced a new way that exploits the Transformer encoders to address the fire prediction task using time-series values collected from multiple sensors. By empirical results, we showed that the traditional machine learning models are better than deep learning models if the given dataset is simple. Our model exhibited better performance for the complex dataset (i.e., NIST dataset), which indicates that it has a greater potential for real-world applications that probably have complex patterns and scenarios. Nevertheless, there is big room for performance improvement, especially in the NIST dataset. We believe that our work contributes to not only fire prediction but also other various prediction tasks, such as risk prediction for patients or health-care services. We also believe that this work may contribute to developing fire surveillance system using autonomous vehicles or drones. We are working on collecting more data from sensors, and the dataset will be manually annotated for the fire prediction task. We will also develop pre-trained Transformer-based models with a large dataset, and investigate the combination of Transformer and other models for more performance improvement.

## Figures and Tables

**Table 1:** Dataset statistics.

		File Names of the Original Dataset	Number of Samples
NIST	Train	sdc02, sdc08, sdc11, sdc12, sdc13, sdc30, sdc31, sdc34, sdc35,	normal:fire = 873:586
		sdc36, sdc37, sdc38, sdc39, sdc40, sdc41	
	Test	sdc07, sdc09, sdc10, sdc14, sdc15, sdc32, sdc33	normal:fire = 112:113
Pascal	Train	Indoor Fire Dataset with Distributed Multi-Sensor Nodes	normal:nuisance:fire = 3813: 199:578
	Test		normal:nuisance:fire = 416:21:66

**Table 2:** Experimental results with $D_{NIST}$, where FPR and TPR represent the false positive rate and true positive rate of the ‘fire’ class, respectively.

Model	Accuracy	FPR	TPR	TPR-FPR
SVM	0.6281	0.6964	0.9499	0.2535
RF (200 estimators)	0.6400	0.4256	0.7050	0.2794
GRU (4 layers)	0.6089	0.7679	0.9823	0.2144
Transformer encoder stack (1 layer)	0.6948	0.4911	0.8791	0.3880
Transformer encoder stack (2 layer)	0.6518	0.6101	0.9115	0.3014
Transformer encoder stack (4 layer)	**0.6963**	0.4405	0.8319	**0.3914**
Transformer encoder stack (6 layer)	0.6444	0.5119	0.7994	0.2875
Transformer encoder stack (8 layer)	0.6652	0.2798	0.6106	0.3308

**Table 3:** Experimental results with $D_{Pascal}$, where FPR and TPR represent the false positive rate and true positive rate of the ‘fire’ class, respectively.

Model	Accuracy	FPR	TPR	TPR-FPR
SVM	0.9616	0.0091	0.8125	0.8034
RF (200 estimators)	**0.9907**	0.0023	0.9688	**0.9665**
GRU (2 layers)	0.9607	0.0334	0.9167	0.8833
Transformer encoder stack (1 layer)	0.9734	0.0091	0.8594	0.8503
Transformer encoder stack (2 layer)	0.9766	0.0091	0.8958	0.8867
Transformer encoder stack (4 layer)	0.9726	0.0091	0.8698	0.8607
Transformer encoder stack (6 layer)	0.9766	0.0114	0.8906	0.8792
Transformer encoder stack (8 layer)	0.9744	0.0099	0.8802	0.8703

## Data Availability

All datasets are publicly available on Web (accessed on 4 April 2024).
